# Examining Ethnic Exposure through the Perspective of the Neighborhood Effect Averaging Problem: A Case Study of Xining, China

**DOI:** 10.3390/ijerph17082872

**Published:** 2020-04-21

**Authors:** Yiming Tan, Mei-Po Kwan, Zifeng Chen

**Affiliations:** 1 Guangdong Provincial Key Laboratory of Urbanization and Geo-Simulation, School of Geography and Planning, Sun Yat-sen University, Guangzhou 510275, China; tanym06@gmail.com; 2 Department of Geography and Resource Management, and Institute of Space and Earth Information Science, Chinese University of Hong Kong, Shatin, Hong Kong, China; mpk654@gmail.com; 3 Department of Human Geography and Spatial Planning, Utrecht University, 3584 CB Utrecht, The Netherlands; 4 Department of Urban Planning and Design, The University of Hong Kong, Pokfulam, Hong Kong, China

**Keywords:** neighborhood effect averaging problem (NEAP), environmental exposure, geographic context, ethnic groups, uncertain geographic context problem (UGCoP)

## Abstract

An increasing number of studies have observed that ignoring individual exposures to non-residential environments in people’s daily life may result in misleading findings in research on environmental exposure. This issue was recognized as the neighborhood effect averaging problem (NEAP). This study examines ethnic segregation and exposure through the perspective of NEAP. Focusing on Xining, China, it compares the Hui ethnic minorities and the Han majorities. Using 2010 census data and activity diary data collected in 2013, the study found that NEAP exists when examining ethnic exposure. Respondents who live in highly mixed neighborhoods (with high exposures to the other ethnic group) experience lower activity-space exposures because they tend to conduct their daily activities in ethnically less mixed areas outside their home neighborhoods (which are more segregated). By contrast, respondents who live in highly segregated neighborhoods (with low exposures to the other ethnic group) tend to have higher exposures in their activity locations outside their home neighborhoods (which are less segregated). Therefore, taking into account individuals’ daily activities in non-residential contexts in the assessment of environmental exposure will likely lead to an overall tendency towards the mean exposure. Using Tobit models, we further found that specific types of activity places, especially workplaces and parks, contribute to NEAP. Ignoring individual exposures in people’s activity places will most likely result in misleading findings in the measurement of environmental exposure, including ethnic exposure.

## 1. Introduction

Geographic context is a key construct for evaluating individual exposures to environmental and social factors (e.g., fresh food markets, pollution, and different social groups). In many studies, researchers have used places of residence to delineate contextual areas or neighborhoods owing to insufficient knowledge about the accurate distributions of the contextual factors in space and time that affect human behaviors. In the last decade or so, however, an increasing number of studies have observed that ignoring individuals’ exposures to non-residential contexts in their daily lives may result in misleading findings in the study of environmental exposure [1,2,3,4,5].

While these studies underpinned the necessity to accurately delineate contextual areas that also include the locations of people’s daily activities beyond residence-based neighborhoods, rather than relying solely on residence-based neighborhoods, some recent studies have taken a further step by highlighting the patterns of estimation errors and discovering a specific reason that contributes to such errors [6,7,8]. Kwan [1] synthesized these findings and articulated the issue as the neighborhood effect averaging problem (NEAP), which is the problem that people’s daily mobility will bring their mobility-dependent exposures (e.g., noise and air pollution) closer to the average of the participants or population of a study area. She postulated that using residence-based neighborhoods to estimate individuals’ mobility-dependent exposures will lead to overestimations of the exposures of individuals who have high residence-based exposures and underestimations of the exposures of those who have low residence-based exposures.

The NEAP is a fundamental methodological issue in studies of the neighborhood effect and environmental exposure. It suggests that if people’s daily activities are ignored, mobility-dependent exposures could be overestimated for people with high residence-based exposures and underestimated for people with low residence-based exposures. Since its articulation in 2018, there are only a few empirical studies on the NEAP, and all of these studies focused on individual exposure to air pollution [9,10,11]. More empirical evidence on the NEAP based on examining other types of mobility-dependent exposures is thus sorely needed.

Recently, there has been a rise in scholarly interest in framing socio-spatial segregation in terms of the locations where individuals conduct their everyday activities, and thus have opportunities to encounter other social groups [12,13,14]. This emerging line of inquiry assumes that the possibility of inter-group interactions could be enhanced owing to the co-presence of individuals from different social groups at the locations of their daily activities. These studies found that residents living in highly segregated neighborhoods are likely to work or perform other daily activities in relatively less segregated areas [15,16,17]. Such observations suggest that a person’s exposure to individuals of other social or racial/ethnic groups may be considered as a type of mobility-dependent exposure, and the NEAP may also exist when studying racial/ethnic segregation.

Aligning with the recent examinations of individual activity spaces and racial/ethnic segregation [12,13,14,15,17,18], this study conceives racial/ethnic segregation as a type of individual exposure to the social environment [13]. Under this conceptualization, a person’s exposure to people of other racial/ethnic groups is treated as the person’s racial/ethnic exposure and is used to capture the extent to which he/she experiences racial/ethnic segregation. After conceiving segregation through the lens of environmental exposure, the study then investigates whether the NEAP exists in the analysis of individual exposures to other racial/ethnic groups. Specifically, it seeks to examine whether people living in highly segregated neighborhoods (e.g., with low exposures to other racial/ethnic groups) tend to have higher exposures to other racial/ethnic groups during their daily activities in other areas outside their residential neighborhoods (which are more likely to be less segregated). Similarly, people living in a highly integrated neighborhood (e.g., with high exposures to other racial/ethnic groups) may tend to have lower ethnic exposures during their daily activities in areas outside their residential neighborhoods (which are more likely to be less integrated and more segregated). Next, the study analyzes which types of activity places (e.g., workplaces, shops, and religious sites) contribute more to the NEAP owing to participants’ different exposures in such types of places when compared with residence-based exposures.

We conduct a study in Xining, China, and focus on the ethnic minority group (Hui) and the majority group (Han) in the city. The data we use include the 2010 population census (for quantifying neighborhood ethnic composition) and activity diary data collected in 2013 from 850 Han respondents and 215 Hui respondents (for capturing individual activity locations). Using conventional residence-based exposure measures, we first identify the neighborhoods with high exposures to ethnic minorities/majorities (e.g., highly mixed neighborhoods) and those with low exposures (e.g., highly segregated neighborhoods). Then, using activity-based exposure measures as a means to capture people’s mobility-dependent exposures to the other ethnic groups, we compare the participants’ activity-based and residence-based exposures (particularly those living in the highly mixed or highly segregated neighborhoods). For each individual, we calculate the deviation between the overall activity-based and residence-based exposures to assess the NEAP. Tobit regression analysis is then conducted to examine which types of activity places (e.g., workplaces, shopping facilities, and religious sites) contribute more to the NEAP owing to the different exposures in such places when compared with residence-based exposures.

This study seeks to enhance our understanding of the NEAP based on empirical evidence on the socio-spatial segregation of ethnic groups in a Chinese city. The findings could provide evidence on whether the NEAP is a serious methodological issue in the studies of individual exposure to the social environment, which includes racial or ethnic composition. Through identifying which types of activity places (e.g., workplaces, shops, and religious sites) contribute more to the NEAP, we draw attention to several types of activity places (especially workplaces and parks and green spaces), because ignoring individual exposures in these activity places will highly likely lead to erroneous results in the measurement of ethnic exposure. The findings can also inform public policies concerning the need to address inter-group encounters in people’s activity places (e.g., shopping facilities) and mitigate prejudice and stigma between ethnic groups in these places of encounters outside people’s residential neighborhoods.

## 2. Literature Review

### 2.1. From the Uncertain Geographic Context Problem (UGCoP) to the Neighborhood Effect Averaging Problem (NEAP)

The uncertain geographic context problem (UGCoP) is a fundamental methodological problem confronting studies on the neighborhood effect and individual exposures. It has attracted considerable scholarly attention in recent years. The UGCoP refers to the problem that findings about neighborhood effects on individual behaviors or outcomes may be erroneous because such effects may be obscured by the inappropriately delineated contextual areas or neighborhoods [2,19]. Several studies have provided evidence on the UGCoP by showing that using residential neighborhoods to delineate contextual areas while ignoring daily human mobility may result in erroneous findings in the evaluation of environmental exposures [3,4,5,20,21]. Some recent studies extended this interest to the empirical context of urban China [22,23]. For instance, using a Chinese city as a case study, Tan et al. [24] delineated two types of contextual units for deriving built-environment variables: one is merely based on the residential neighborhood and the other considers both the residential neighborhood and people’s activity spaces. Through comparison, the study found that geographic contexts based only on people’s residential neighborhoods may lead to misleading results, such as overestimating the contextual effects of the residential neighborhoods and underestimating those of other activity places.

**Figure 1:**
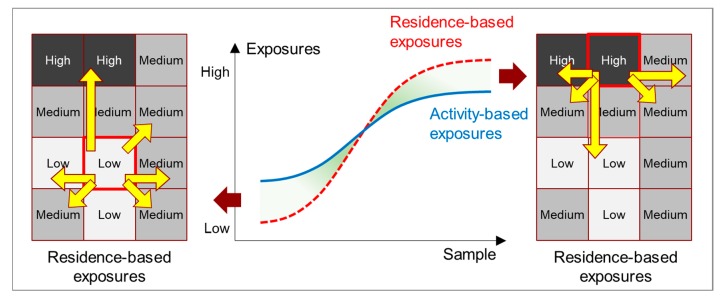
Conceptual diagram of the neighborhood effect averaging problem (NEAP).

The aforementioned studies underpinned the necessity to accurately delineate contextual areas by incorporating human mobility rather than by relying on residence-based neighborhoods. More recently, Kwan [1] articulated another methodological issue called the neighborhood effect averaging problem (NEAP), which can be considered a specific manifestation of the UGCoP. The NEAP suggests that an individual who lives in a neighborhood with a very high level of an environmental factor tends to have lower exposures to such a factor when conducting activities in other areas outside of his/her residential neighborhood (because other areas in the city are much more likely to have lower levels of such factor), and vice versa. Taking into account individuals’ daily mobility in the assessment of environmental exposure will thus likely lead to an overall tendency towards the mean exposure. Further, using residence-based neighborhoods to estimate individual exposures will lead to biased estimations by exaggerating the disparities in exposures among individuals (Figure 1).

Several recent studies not only provided strong evidence that the NEAP exists when examining mobility-dependent exposure but also showed that different social groups may be associated with different levels of the NEAP [6,7,8,9,10]. For instance, using mobile phone data in Belgium, Dewulf et al. [6] found that individuals with low residence-based NO_2_ exposures witness a considerable increase in NO_2_ exposure when their travel patterns are considered. This is also true the other way around; individuals with high residence-based NO_2_ concentrations experience a decrease in NO_2_ exposure after incorporating their travel patterns. In the study of exposure to air pollution in Israel, Shafran-Nathan et al. [7] found that the distributions of the differences between outdoor exposure and exposure at home are symmetric around zero. This suggested that, for each individual, there is an almost equal probability that residence-based exposure will be either higher or lower than activity-based exposure.

Similar findings are obtained by Yu et al. [8] in their study of individual exposure to air pollution in Shenzhen: the residence-based exposure estimates have greater variability than the mobility-based exposure estimates, and individuals with high residence-based exposures tend to have lower mobility-based exposures, while individuals with low residence-based exposures tend to have higher mobility-based exposures. X. Ma et al. [9] assessed the real-time air pollution exposure and residence-based exposure of the residents in a high-pollution neighborhood in Beijing. They found that, owing to the NEAP, participants may have lower exposures due to their daily mobility. In a study of Los Angeles, Kim and Kwan [10] found that the NEAP exists when assessing individual exposures to air pollution and that high-income, employed, younger, and male participants (when compared with low-income, non-working, older, and female participants) experienced higher levels of neighborhood effect averaging because of their higher levels of daily mobility.

These studies elucidated the need to address the NEAP in studies of air pollution [1]. However, more empirical evidence is needed to investigate whether the NEAP also manifests in the studies of other types of mobility-dependent exposures. Several recent studies suggested that the NEAP may also exist in the study of socio-spatial segregation and ethnic exposure, in the way that residents living in highly segregated neighborhoods tend to conduct daily activities in other areas out of their home neighborhoods that are very likely to be less segregated when compared with those in their residential neighborhoods, and vice versa [16,17].

### 2.2. Activity-Based Segregation and Exposure

As a well-researched topic that covers distinct disadvantaged social groups (e.g., ethnic minorities and low-income residents), socio-spatial segregation has long been understood as spatial separation or isolation of residential locations [25,26,27,28]. In recent decades, there has been a rise of scholarly interest in framing socio-spatial segregation in terms of locations where individuals conduct everyday activities, and thus have opportunities to encounter other social groups, based on the assumption that such encounters are conducive to higher levels of inter-group interactions [12,13,14,15,29]. High exposures to different social groups are considered as low levels of socio-spatial segregation and have a high potential for social interactions in this literature [30].

Schnell and Yoav [31] approached this issue by incorporating individuals’ isolation from members of other social groups in people’s everyday-life activity spaces into common segregation indices. Wong and Shaw [12] advanced the conventional residential segregation measure by highlighting individuals’ activity spaces. Their measure reflects how individuals are exposed to other population groups that are present in their daily activity spaces. Using a similar approach, Krivo et al. [15] examined how demographic characteristics (e.g., the percentages of the low-income, unemployed residents, and female-headed families) of the neighborhoods where people conduct daily activities vary by income and ethnic groups. Shareck et al. [18] observed the existence of social isolation (e.g., educational inequalities) in both residential areas and activity locations. Focusing on the segregation between public and private housing residents, Wang and Li [14] adopted a similar perspective to examine individuals’ exposure to other social groups in their activity spaces. They find that, compared with private housing residents, public housing dwellers are more likely to encounter people similar to themselves. Using a Chinese city as a case study, Tan et al. [32] measured and compared the ethnic exposure in the activity spaces of individuals in different social groups. According to their observation, residents of different social groups, even when residing in neighborhoods that are similar in ethnic composition, are exposed to significantly different ethnic contexts in their out-of-home activity locations. These studies uncovered substantial differences between residence-based and activity-based segregation.

The present study, joining this recent literature on activity spaces and segregation, seeks to enhance our understanding of the socio-spatial segregation of two ethnic groups (Han and Hui) in the Chinese city of Xining through the perspective of the NEAP. It conceptualizes racial/ethnic segregation as a type of individual exposure to the social environment, where a person’s exposure to people of other racial/ethnic groups is considered the person’s racial/ethnic exposure, which is then used to capture the extent to which he/she experiences racial/ethnic segregation. After conceiving segregation through the lens of environmental exposure, the study then investigates whether the NEAP exists in the analysis of individual exposures to other racial/ethnic groups. Specifically, the study seeks to examine whether people living in highly segregated neighborhoods (e.g., with low exposures to other racial/ethnic groups) tend to have higher exposures to other racial/ethnic groups during their daily activities in other areas outside their residential neighborhoods (which are more likely to be less segregated), and vice versa. It also attempts to examine which types of activity places contribute more to the NEAP owing to the different exposures in such types of places when compared with residence-based exposures.

## 3. Study Area, Data, and Methods

### 3.1. Study Area and Data

**Figure 2:**
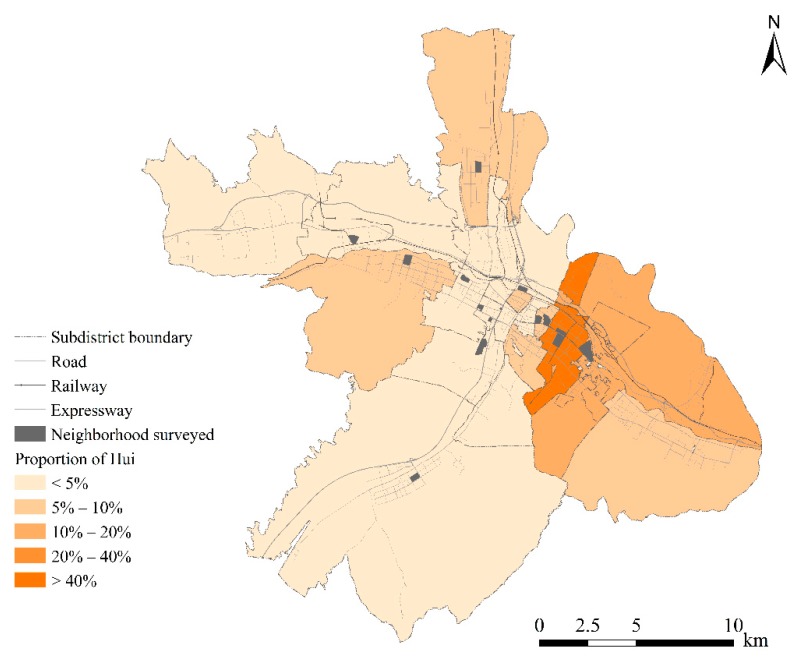
The study area.

There are officially 56 ethnic groups in China, including a majority group (e.g., Han) and 55 minority groups. The latter in total accounted for 8.5% of the national population in 2010 (according to the population census). This study was conducted in Xining, a city in China with high ethnic diversity. The city has a population of 2.37 million (based on the 2018 Annual Report of Economic and Social Development [33]). Ethnic minorities (including Hui, Tibetan, Tu, and Mongolian, among others) constitute 25.8% of the city’s population. Among the ethnic minorities in Xining, the Hui are the largest minority group, and comprise 16.2% of the city’s population. The Hui minorities predominantly (over 70%) reside in the central and eastern parts of the urban area of Xining (Figure 2). The Hui are Muslims who integrated their Islamic characteristics with some characteristics of Chinese lifestyles. This study focuses on the Hui and the Han ethnic groups in the city. A recent study found that the ethnic composition in the neighborhoods where people conduct everyday activities is considerably different between the two groups, despite the less segregated residential patterns in the city [32].

Two datasets are utilized in this study. One is the 2010 subdistrict-level census data of Xining that provide information about the ethnic composition in each of 28 subdistricts of the city (each subdistrict has a population of 428,000 on average). The other one is the individual activity diary dataset collected through a residence-based questionnaire survey in September 2013. The survey adopted a two-stage sampling strategy to maximize representativeness regarding the diversity of residential neighborhoods (a residential neighborhood is subordinate to a subdistrict and houses 1500–3000 residents on average). First, 15 residential neighborhoods were selected from the 28 subdistricts to represent the variety of locations, years of construction, as well as the percentages of Hui residents (Figure 2). Among these 15 neighborhoods, two were developed in the 1980s near the city center (e.g., around the crossing of the horizontal and vertical expressways), eight were developed after 2000 (or even after 2010) and were located between the center and periphery, and five were located in the periphery of the study area. These neighborhoods were also diverse by the proportions of Hui. Two neighborhoods were selected from subdistricts where over 40% of residents were Hui, while eight neighborhoods were chosen from Han-dominated subdistricts where the Hui people constituted only 5% or fewer. Second, we obtained the address list from the resident committee (*shequ juweihui*) of each residential neighborhood. The address list presented all dwelling units in each building of the neighborhood as a basis of systematic sampling. An initial systematic sampling was conducted to select 100 households from each neighborhood. We compared the ethnic composition of these 100 selected households in each neighborhood with the actual ethnic composition of the neighborhood (which was provided by the resident committee), and randomly deleted/added households until the former became equal to the latter. In each household, both female and male household heads were invited to participate in the survey. Therefore, about 200 respondents were recruited in each neighborhood. Each respondent was asked to complete a questionnaire of socio-demographic attributes and a 48 h activity diary (including a Sunday representing a weekend and a Monday representing a weekday). The respondent was asked to report the information of each activity that she or he undertook within the 48 h survey period, including the type of activity (based on 16 options), timing and duration of the activity, address, and type of activity location (e.g., home or workplace). All the addresses are geocoded into longitude/latitude coordinates. A total of 2598 individuals returned the questionnaire with valid answers for socioeconomic attributes. However, some participants did not provide complete records for the activity diary needed for the study. Our research is thus limited to the 1065 respondents who provided complete information about their out-of-home activities. These 1065 respondents include 850 Han residents and 215 Hui residents (constituting 20.2% of the sample, which is close to the percentage of the Hui minorities in the census data (e.g., 16.3%)). As shown in Table 1, the socioeconomic attributes of the final sample (1065 respondents) are very similar to those of the original sample (2598 respondents), indicating that excluding 1533 respondents only led to limited systematic bias in the data.

The socioeconomic attributes of the Hui and Han respondents are also presented in Table 1. The age distribution and ratio of females to males are quite similar between the two ethnic groups without a significant difference. In total, 62.3% of the Hui respondents are low-income residents, with an average monthly income of 2000 CNY (Chinese Yuan), or below. An overwhelming majority (81.2%) of the Han participants are local residents with local hukou. Compared with the Han participants, a significantly higher proportion of the Hui participants are migrants (31.2%). Moreover, 71.6% of the Hui respondents have a low educational level (e.g., middle school or below), while the proportions of the Han respondents with a medium education level (e.g., high school) or higher (e.g., college or above) are significantly larger than those of Hui respondents. More than half of the Han residents are full-time workers. Among the Hui residents, only 24.8% work full time, while 37.4% have part-time jobs such as street vendors.

### 3.2. Measuring Residence-Based and Activity-Based Ethnic Exposure

Individuals’ ethnic exposure is quantified as the ethnic composition (derived from census data) at specific locations. Two types of ethnic exposure are investigated in this study. One is residence-based exposure, representing exposure in the neighborhood where an individual resides. The other one is activity-based exposure, representing exposure in the neighborhoods where the person conducts everyday activities. Following the method presented in previous studies [12,32], we first measure residence-based exposure to capture the potentials of inter-group interaction in participants’ residential places. We adopt subdistricts as the spatial unit to measure residence-based exposure (as well as activity-based exposure), because the 28 subdistricts cover the whole study area so that we can capture the spatial variation of residence-based exposure across the study area. The Hui and Han ethnic groups (which together comprise an overwhelming proportion of the population in Xining) are denoted as Groups A and B, respectively. In subdistrict *i*, the exposure of Group A to Group B and that of Group B to Group A are formulated as Equations (1) and (2), respectively.

(1) $$E_{i^{*}ab}=\frac{a_{i}\sum_{j}c_{ij}b_{j}}{a_{i}\sum_{j}b_{j}},$$

(2) $$E_{i^{*}ba}=\frac{b_{i}\sum_{j}c_{ij}a_{j}}{b_{i}\sum_{j}a_{j}},$$

where $a_{i}$ and $b_{i}$ are the population of Groups A and B in subdistrict *i* (derived from census data), respectively. The denominator denotes the potential encounters between $a_{i}$ with all individuals in Group B in the study area, and vice versa. $c_{ij}$ denotes the element of the binary adjacency matrix. This component is necessary because, according to Morrill [34], “the pattern of differences in the proportions of minority across all adjacent boundaries” (p. 34) should be taken into account in the measurement of exposure. The value of “1” for $c_{ij}$ indicates that the corresponding *i* and *j* subdistricts are neighbors (e.g., the two subdistricts share part of their borders), while the value of “0” indicates otherwise (it is noteworthy that *i* can be equal to *j*, and $c_{ii}$ equals to 1). The residence-based exposure is standardized to a range of 0 (indicating no exposure or perfect segregation) to 1 (indicating perfect exposure or no segregation).

The residence-based exposure measures of Groups A or B are then extended to activity-based exposure measures for each individual of the respective group. For each individual, we identify the subdistricts where she/he performs daily activities based on the activity diary data. The exposure in each of these subdistricts is weighted by her/his activity duration in the subdistrict. We then average all these time-weighted exposures into a single indicator, representing the individual’s activity-based exposure in all of the non-residential places or neighborhoods.

### 3.3. Measuring the Extent of Neighborhood Effect Averaging

To measure the extent of neighborhood effect averaging for the participants, we further develop a series of deviation indexes based on the absolute value of the differences between activity-based and residence-based exposures (while the non-absolute value will also be examined and presented in Table 2. These deviation indexes include the following: (1) the overall deviation index, denoted as *D* (for all out-of-home activity places); (2) the deviation index for each activity place (e.g., a restaurant), denoted as $D_{i}$; and (3) the deviation index for each *type* of activity place (e.g., all restaurants), denoted as $D_{p}$.

The overall deviation index captures the differences between activity-based exposures in all out-of-home activity places and residence-based exposures, specified as follows.

(3) $$D=\left|E_{\bar{activity}}-E_{residence}\right|,$$

where $E_{\bar{activity}}$ and $E_{residence}$ denote an individual’s activity-based exposures in all out-of-home activity places and residence-based exposures, respectively. Using the same approach, we develop a deviation index for each out-of-home activity place (e.g., a restaurant), specified as follows.

(4) $$D_{i}=\left|E_{activity_{i}}-E_{residence}\right|,$$

where $E_{activity_{i}}$ is the exposure of individual *k* in an activity place *i*.

Next, we take a further step to develop a deviation index for each *type* of activity places (e.g., all restaurants). The aforementioned deviation index for each activity place ($D_{i}$) is weighted by the corresponding activity duration. The time-weighted $D_{p}$ of several activity places that belong to the same type are then averaged into a single index, specified as follows.

(5) $$D_{p}=\frac{\left|E_{activity_{1}}^{p}-E_{residence}\right|t_{1}+\left|E_{activity_{2}}^{p}-E_{residence}\right|t_{2}\cdots +\left|E_{activity_{i}}^{p}-E_{residence}\right|t_{i}}{t_{1}+t_{2\;}+\cdots +t_{i}},$$

where $D_{p}$ represents the deviation index for activity place of type *p* for an individual, $E_{activity_{i}}^{p}$ denotes an individual’s exposure in an activity place *I*, and $t_{i}$ represents the activity duration in place *i*.

### 3.4. One-Limit Censored Tobit Model Analysis

Next, the study proceeds to examine which types of activity places (e.g., workplaces, shops, and religious sites) contribute more to the NEAP owing to the different exposures in such types of places when compared with residence-based exposures. We attempt to predict the effects of exposures in different types of out-of-home activity places on the overall deviation index (*D*), after controlling for participants’ socioeconomic attributes. For individuals who conduct all of their out-of-home activities in the same subdistrict as their home subdistrict with short travel distances, the overall deviation index values are zero, as these individuals have the same activity-based exposures and residence-based exposures. This results in a considerable number of zero observations (53 for the Hui respondents and 202 for the Han respondents) for the dependent variable. Therefore, we adopt two one-limit censored Tobit models (including Model 1 for the Han subsample and Model 2 for the Hui subsample), which employ a discrete-continuous type of modeling framework and are formulated as follows:

(6) $$y_{i}=f\left(x\right)=\{\begin{array}{cc} y_{i}^{*}, & \mathrm{if}\;y_{i}^{*}>0\\ 0, & \mathrm{if}\;y_{i}^{*}\;\leq \;0 \end{array},$$

(7) $$y_{i}^{*}=\;\beta x_{i}+\gamma SES+u_{i},\;\;\;\;u_{i}\sim N\left(0,\sigma^{2}\right),$$

where $y_{i}$ denotes the dependent variable, that is, the overall deviation index (*D*). $x_{i}$ represents a set of independent variables that measure the deviation indices in different types of activity places ($D_{p}$), including workplaces, relatives’ homes, shops, restaurants, parks and green spaces, hospitals, and religious sites. *SES* represents a set of variables of participants’ socioeconomic status, including gender, age, education, *Hukou*, employment status, and income. These two one-limit censored Tobit models are estimated using Stata’s Tobit module. The following sections present the results based on the deviation indexes and the models.

## 4. Results

### 4.1. Descriptive Analysis

This section presents a descriptive analysis to investigate the NEAP in the ethnic exposures in Xining. Table 2 shows the distribution of the differences between activity-based and residence-based exposures for the participants ranked by residence-based exposures. The respondents are categorized into five quintiles according to their residence-based exposures. Overall, the differences are negative in the first three quintiles and positive in the fourth and fifth quintiles. Respondents of the first quintile (e.g., living in neighborhoods with very low residence-based exposures) have the largest positive differences, indicating that their activity-based exposures tend to be substantially higher than their residence-based exposures. By contrast, respondents of the fifth quintile (e.g., living in neighborhoods with very high residence-based exposures) have the largest negative differences, indicating that their activity-based exposures tend to be substantially lower than their residence-based exposures. Meanwhile, respondents of the third quintile (e.g., living in neighborhoods with medium residence-based exposures that are around the mean value) have the smallest differences. This result suggests that smaller differences between activity-based and residence-based exposures are found for respondents who live in neighborhoods with medium residence-based exposures when compared with those who live in highly mixed or highly segregated neighborhoods. We take a further step by conducting a paired-sample *t*-test to compare the activity-based and residence-based exposures in each quintile. The result shows that for the respondents of the first and second quintiles (e.g., low residence-based exposures), as well as those of the fourth and fifth quintiles (e.g., high residence-based exposures), the differences between activity-based exposures and residence-based exposures are statistically significant (at the 0.01 level). Meanwhile, there is no significant difference between activity-based and residence-based exposures for the respondents of the third quintile.

These patterns in the differences between activity-based and residence-based exposures indicate that the NEAP exists in the ethnic exposures of the participants and that our measures of activity-based exposures can capture the mobility-dependent exposures of the participants. These results are corroborated by the results of an analysis of variance (ANOVA) of the overall deviation indices (*D*) in Table 3. The ANOVA uncovers significant variations (at the 0.01 level) in the overall deviation indices among these five quintiles. The mean overall deviation index of the first quintile (e.g., lowest residence-based exposures) is larger than that of the second quintile; the mean the overall deviation index of the fifth quintile (e.g., highest residence-based exposures) is larger than that of the fourth quintile. On average, respondents of the third quintile have the lowest overall deviation indices. These results are consistent with the findings from Table 2, indicating that respondents who live in neighborhoods with medium residence-based exposures tend to have small overall deviation indices, while those who live in neighborhoods with very high or very low residence-based exposures tend to have large overall deviation indices.

**Figure 3:**
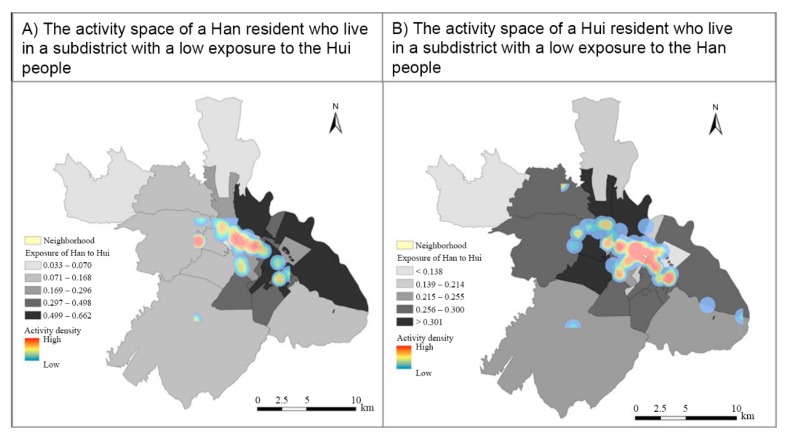
Examples of a Han resident and a Hui resident: (**A**) the activity space of a Han resident who lives in a subdistrict with low exposure to the Hui people; (**B**) the activity space of a Hui resident who lives in a subdistrict with low exposure to the Han people.

These findings are consistent with the hypothesis of the NEAP; that is, activity-based ethnic exposures show an overall tendency towards the mean value of the participants when compared with their residence-based ethnic exposures. Respondents who live in neighborhoods with high ethnic exposures (ethnically mixed neighborhoods) tend to experience lower levels of exposure in their non-residential activity locations. By contrast, respondents who live in neighborhoods with low exposures (highly segregated neighborhoods) tend to experience higher exposures in their non-residential activity locations. These tendencies suggest that the former tend to travel to and perform activities in areas that are likely to have lower ethnic exposures when compared with their residential neighborhoods, while the latter, similarly, tend to travel to areas that are likely to have higher ethnic exposures than their residential neighborhoods. These tendencies are well illustrated by the two examples presented in Figure 3. These two examples are a Han resident who lives in a subdistrict with low exposure to the Hui people, and a Hui resident living in a subdistrict with low exposure to the Han people. As shown in the figure, these two respondents both perform their daily activities in subdistricts with higher exposures to the other ethnic group when compared with their residential subdistricts.

### 4.2. Modeling Results

This section presents the results of the one-limit censored Tobit models (Model 1 for the Han subsample and Model 2 for the Hui subsample, see Table 4). For the Han respondents (Model 1), six out of the seven types of activity places (e.g., except religious sites) have significant impacts on the dependent variable (e.g., the overall deviation index), suggesting that the overall deviation index (*D*) can be decomposed to the deviation indices ($D_{p}$) for workplaces, relatives’ homes, shops, restaurants, parks and green spaces, as well as hospitals. Meanwhile, the deviation index for religious sites does not significantly contribute to the overall deviation index. This is probably because very few Han residents visit religious sites (especially mosques) in their everyday life (Table 4). On average, exposure to religious sites only constitutes a negligible part of the overall activity-based exposure for the Han majority group in the study area.

Next, the study attempts to examine what types of activity places contribute more to the overall deviation index. In linear regression models, the contribution of one independent variable to the dependent variable can be evaluated based on the standardized regression coefficient. Because of its nonlinearity assumptions, however, there is no equivalence of the standardized regression coefficients in the Tobit model. Nevertheless, the *t* ratios in the Tobit model could provide information on the contribution of a given independent variable to the dependent variable. This study will thereby focus on the *t* ratios to compare the contributions of different types of activity places to the dependent variable. Among the six types of activity places that have significant impacts on the dependent variable (e.g., the overall deviation index), the deviation index of workplaces has the largest *t* ratio, followed by the deviation index of shops and of parks and green spaces. This indicates that the deviation indices of workplaces, shops, and parks and green spaces are the largest three contributors to the overall deviation index. Thus, for the Han respondents, neighborhood effect averaging predominantly arises from their non-residential activities in workplaces, shops, as well as parks and green spaces.

Meanwhile, most of the socioeconomic variables are not significantly associated with the dependent variable of the overall deviation index except for “*Hukou*: Local”. The latter is found to be significantly and positively related to the overall deviation index, suggesting that there are large deviations between the activity-based and residence-based exposures for local residents of the Han ethnic group. This is likely because local Han residents have better mobility than migrant Han residents, and thus have a higher probability to travel to various places and be exposed to other ethnic groups. In Chinese cities, social differences tend to be closely associated with mass migration and the *hukou* system, which ties welfare entitlement to a person’s *hukou* status and discriminates against migrants (e.g., non-local *hukou* holders) from local residents. It is well documented in the literature that the *hukou* system renders the migrant population more disadvantaged than local residents in daily mobility and spatial access to public facilities and services [35].

With regard to the Hui respondents (Model 2), six out of the seven types of activity places (e.g., except restaurants) have significant impacts on the dependent variable, indicating that the overall deviation index (*D*) can be decomposed to the deviation indices ($D_{p}$) for workplaces, relatives’ homes, shops, parks and green spaces, hospitals, as well as religious sites. Unlike the Han ethnic group, many Hui residents are religious and regularly visit mosques on a daily basis. Hence, some Hui residents living in mixed neighborhoods (with high exposures to other ethnic groups) have fewer opportunities to encounter Han people in their non-residential activity locations because they spend a considerable amount of their time in mosques where Han residents rarely visit. Interestingly, the deviation index for restaurants does not significantly contribute to the overall deviation index for the Hui people. A possible explanation is that there are few restaurants in the study area that provide *halal* food (e.g., religiously permissible) compared with other types of restaurants. As most Hui residents prefer *halal* food, only a few Hui residents dine out in restaurants and most dine at home (Table 5). As a result, their exposure to other ethnic groups in restaurants only constitutes a negligible part of the overall activity-based exposure of the Hui residents of the study area.

Again, we use the *t* ratios to compare the contributions of different types of activity places to the dependent variable. Among the six types of activity places that have significant impacts on the dependent variable (e.g., the overall deviation index) for the Hui residents, workplaces, parks and green spaces, as well as religious sites are found to be the largest three contributors to the overall deviation index, as indicated by their large *t* ratios. Therefore, for Hui residents, neighborhood effect averaging predominantly arises from their non-residential activities in workplaces, parks and green spaces, as well as religious sites.

As for the socioeconomic determinants of the overall deviation index, only “Employment: Full-time job” has a significant negative influence the overall deviation index of the Hui residents. This negative determinant implies that the Hui respondents who work full time have less deviation between their activity-based and residence-based exposures when compared with those who work part-time, have retired, or are unemployed. Among the Hui respondents, only 24.8% work full time, while 37.4% have part-time jobs such as street vendors (Table 1). Compared with full-time workers, those who work part-time, have retired, or are unemployed (especially the street vendors) experience fewer space-time constraints from their work-related activities, and thus have more opportunities to travel around to different neighborhoods and be exposed to different ethnic groups. Therefore, full-time workers among the Hui residents are more likely to have similar activity-based and residence-based exposures, in contrast to other Hui residents.

## 5. Conclusions

On the basis of a case study of Xining (China) that compared the ethnic exposures of the Hui ethnic minorities and the Han majorities, this study engages the recent debate over the limitations of the neighborhood effect and examines the neighborhood effect averaging problem (NEAP) in the measurement of racial/ethnic segregation. The analysis yielded several important findings. Compared with residence-based exposures, activity-based exposures of the participants show an overall tendency towards the mean value. Respondents who live in highly mixed neighborhoods (with high exposures to other ethnic groups) experience lower activity-based exposures to other ethnic groups because they conduct their daily activities in less ethnically mixed areas when they travel out of their residential neighborhoods. By contrast, respondents who live in highly segregated neighborhoods (with low exposures to other ethnic groups) tend to have higher exposures in their non-residential activity locations. Meanwhile, smaller differences between activity-based and residence-based exposures are found for the respondents who live in neighborhoods with medium residence-based exposures, when compared with those who live in highly mixed or highly segregated neighborhoods.

These findings highlight a nuanced form of socio-spatial segregation confronting contemporary cities in China and other countries across the world. Different social groups tend to have different daily-life patterns in space and time [13]. Ethnic minorities, even when sharing the same residential environment with the majorities, may neither use facilities in their residential and non-residential neighborhoods as much as the majorities, nor use them at the same time as the majorities. Owing to the diverse space–time activity patterns, living in the same residential neighborhood does not necessarily mean similar use of spaces or meaningful cross-group interactions. Therefore, using residence-based neighborhoods to estimate individual exposures to other ethnic groups (or racial/ethnic segregation) may lead to the overestimation of exposures for people with high residence-based exposures and underestimation of exposures for people with low residence-based exposures, as postulated by the NEAP.

Using one-limit censored Tobit models, the study further identified which types of activity places contribute more to the NEAP. For Han residents, the NEAP predominantly arises from their non-residential activities in workplaces, shops, as well as parks and green spaces. For Hui residents, the NEAP predominantly arises from their non-residential activities in workplaces, parks and green spaces, as well as religious sites. These activity places play a vital role in bringing together different ethnic groups that are segregated in their residential neighborhoods or separated from one another even though they live in the same residential neighborhood. We highlight the importance of these activity places (especially workplaces and parks and green spaces) because ignoring people’s exposures in these activity places will highly likely lead to erroneous results in the measurement of ethnic exposure and racial/ethnic segregation. As recent studies suggest, workplaces [36] and open spaces [37] are other areas beyond people’s residential neighborhoods that are important for examining socio-spatial segregation or integration.

To conclude, these findings provide strong evidence on the NEAP as a fundamental methodological issue when examining ethnic exposure or racial/ethnic segregation. While the UGCoP highlights that estimating individual exposures based only on people’s residential neighborhoods may lead to erroneous findings, and thus underpins the need to assess exposures based on people’s daily mobility and activity spaces, the NEAP takes a further step to unpack the specific patterns of such estimation errors, indicating that using residence-based exposure assessment could exaggerate the magnitude of the neighborhood effect on individual behaviors or outcomes. Therefore, the NEAP is a helpful notion for providing a better understanding of the limits of the neighborhood effect and mobility-dependent exposures. It suggests that using people’s activity locations to delineate their socio-geographic contexts rather than their residential neighborhoods can help reduce estimation error and more accurately capture the patterns of ethnic exposure.

This study also advances our understanding of the NEAP in important ways. For instance, although recent studies found strong evidence that the NEAP exists when assessing individual exposures to air pollution, its extent is associated with people’s daily mobility, and certain social groups (e.g., high-income) are associated with higher levels of neighborhood effect averaging because of their higher levels of daily mobility [9,10]; their results are obtained based solely on observing the distribution patterns of the low and high exposures. This study, however, identified specific social and cultural factors (e.g., participation in religious activities and patronage of restaurants affected by the availability of religiously permissible food) that contribute to neighborhood effect averaging in the assessment of ethnic exposures or racial/ethnic segregation.

Further, although the study observed that people living in highly segregated neighborhoods tend to experience higher exposures to other ethnic groups in the locations of their daily activities, this observation does not suggest that such high exposures necessarily mean these people experience less social or racial isolation. As recent studies indicate, even when disadvantaged residents have high exposures to advantaged social groups, their relative isolation and segregation may persist [38]. This is because cross-group contacts or encounters in urban spaces do not necessarily translate into true social integration or respect for difference [39]; rather, spaces of encounters could remake and enact social difference, and such encounters can also expose minorities to discrimination [13,37]. It is thus important to mitigate prejudice between racial/ethnic groups in non-residential places and stigmatization that may reinforce social isolation [40].

The policy implications of this study are twofold. First, for highly mixed neighborhoods, ethnic residential mixing is not necessarily conducive to social integration or cohesion as people may move around and experience different levels of ethnic exposures outside their residential neighborhoods. Therefore, planning and policies should also address the inter-group encounters in people’s activity places (e.g., shopping facilities) outside their residential neighborhoods and their possible outcomes. Second, residents who live in highly segregated neighborhoods (e.g., ethnic minority enclaves) tend to have a higher potential for encountering other ethnic groups during their daily activities outside their residential neighborhoods. Planning and polices should endeavor to mitigate prejudice and stigma between ethnic groups in these non-residential places of encounters in order to promote social integration in the spaces of everyday life.

Several limitations of this study need to be acknowledged. First, exposure in this study is operationalized as potential co-presence within a subdistrict rather than actual encounters or contacts. Second, we did not consider the dynamics of ethnic composition in activity spaces or address the timing of co-presence [17], because we rely on static census data to quantify ethnic composition rather than dynamic population data (e.g., mobile phone data). Third, owing to the limited availability of data, we only used subdistrict-level population census data to measure exposure. There could be variations in exposure within each subdistrict that need to be addressed in future studies using more fine-grained population data. Fourth, owing to the limited spatial sampling of activity diary data, our observations of ethnic exposures are based on data from several selected neighborhoods rather than a sample of neighborhoods that are evenly spread around the entire study area. Future studies should utilize new datasets with larger sample sizes and higher representativeness to derive more reliable evidence. Finally, as discussed above, higher ethnic exposure does not necessarily mean more inter-group social interactions. A more grounded examination is needed to decipher the implications of the NEAP and activity-based exposure for enhancing social integration and minimizing ethnic stigmatization.

## Figures and Tables

**Table 1:** Characteristics of the participants in the sample.

Variables		Final Sample		Han Ethnic Group		Hui Ethnic Group		Sig. ^1^
		Number	%	Number	%	Number	%	
Total		1065	-	850	-	215	-	-
Gender	Female	511	48.0% (49.4%) ^2^	415	48.8%	96	44.7%	0.27
	Male	554	52.0% (50.6%)	435	51.2%	119	55.3%	
Age ^3^	<30	100	9.4% (9.9%)	69	8.1%	31	14.4%	0.01
	30–50	647	60.8% (64.2%)	515	60.6%	132	61.4%	
	>50	318	29.9% (25.9%)	266	31.3%	52	24.2%	
Monthly Income ^4^ (CNY)	<2000	388	36.4% (40.6%)	254	29.9%	134	62.3%	0.00
	2000–5000	580	54.5% (50.3%)	509	59.9%	71	33.0%	
	>5000	97	9.1% (9.1%)	87	10.2%	10	4.7%	
*Hukou* Status	Temporary migrants	227	21.3% (26.5%)	160	18.8%	67	31.2%	0.00
	Local	838	78.7% (73.6%)	690	81.2%	148	68.8%	
Education Attainment	Middle school or below	391	36.7% (35.0%)	237	27.9%	154	71.6%	0.00
	High school	407	38.2% (42.4%)	360	42.4%	47	21.9%	
	College or above	267	25.1% (22.7%)	253	29.8%	14	6.5%	
Employment Status	Full-time job	490	46.0% (43.4%)	438	51.5%	52	24.2%	0.00
	Part-time job or other	212	19.9% (21.0%)	131	15.4%	81	37.7%	
	Unemployed	210	19.7% (17.2%)	187	22.0%	23	10.7%	
	Retired	153	14.4% (18.5%)	94	11.1%	59	27.4%	

^1^ Significant level of the Chi-square test for the Han and Hui ethnic groups. ^2^ Numbers in brackets represent the percentage among the original sample (2598 respondents). ^3^ The variable of age comprises three categories, namely, “<30”, “30–50”, and “>50”. For residents aged 30 or younger by 2013 (the year of questionnaire survey), they were born in the 1980s (or later), which was the post-reform period in China. For residents aged 30 to 50 by 2013, they were born during the 1960s–1980s, which were accepted as the baby boomer years of China. ^4^ The variable of income comprises three categories, namely, “<2000”, “2000–5000”, and “>5000”. The minimum wage standard in Xining was set to 1700 CNY (Chinese Yuan), while the average monthly wage of residents in Xining was approximately 5000 CNY. Therefore, the three income categories (“<2000”, “2000–5000”, “>5000”) can fall into the city’s low-income group, lower-middle-income group, and higher-middle to high-income group, respectively.

**Table 2:** Differences between (and paired-sample *t*-test of) activity-based and residence-based exposures.

Quintile of Residence-Based Exposure	Residence-Based Exposures (Mean)	Activity-Based Exposures (Mean)	Differences between Average Activity-Based and Residence-Based Exposures	Sig. ^1^ (Paired *t*-Test)	Number of Respondents
1st	0.182	0.229	**0.047 ^2^**	0.000	246
2nd	0.236	0.269	**0.033**	0.000	232
3rd	0.297	0.292	−0.005	0.108	229
4th	0.417	0.350	**−0.067**	0.000	188
5th	0.561	0.459	**−0.102**	0.000	193

^1^ Sig.—significance value. ^2^ Number in bold represents significant coefficients at 0.01 level.

**Table 3:** Analysis of variance (ANOVA) for the overall deviation index (*D*).

Quintile of Residence-based Exposure	Mean	Standard Deviation	Number of Respondents
1st	0.053	0.073	246
2nd	0.045	0.057	232
3rd	0.040	0.052	229
4th	0.068	0.077	188
5th	0.116	0.149	193
Sig. ^1^	0.000		

^1^ Sig.—significance value.

**Table 4:** Results of one-limit censored Tobit models.

Variables	Model 1 (Han Sample)			Model 2 (Hui Sample)		
	Coefficient	t	P > |t|	Coefficient	t	P > |t|
*Gender (Ref: Male)*						
Female	−0.01	−0.73	0.47	−0.01	−1.00	0.32
*Age (Ref: >50)*						
<30	0.00	0.17	0.87	0.00	0.28	0.78
30–50	−0.01	−0.45	0.66	0.01	1.52	0.13
*Education (Ref: College or above)*						
Middle school or below	−0.03	−1.72	0.09	0.00	−0.25	0.80
High school	−0.02	−1.46	0.15	0.00	−0.11	0.92
*Hukou (Ref: Temporary migrants)*						
Local	**0.02 ^1^**	**2.10**	**0.04**	0.00	−0.69	0.49
*Employment (Ref: Unemployed)*						
Full-time job	−0.02	−1.35	0.18	**−0.03**	**−2.83**	**0.01**
Part-time job or other	0.02	1.02	0.31	0.00	−0.22	0.83
Retired	−0.02	−0.96	0.34	−0.02	−1.75	0.08
*Income (Ref: >5000)*						
<2000	−0.01	−0.42	0.68	0.01	0.33	0.74
2000–5000	0.00	0.17	0.87	0.01	0.92	0.36
*Deviation index in each type of activity places*						
Workplaces	**0.15**	**15.23**	**0.00**	**0.27**	**14.26**	**0.00**
Relatives’ home	**0.19**	**6.58**	**0.00**	**0.24**	**3.28**	**0.00**
Shops	**0.38**	**11.01**	**0.00**	**0.17**	**2.61**	**0.01**
Restaurants	**0.07**	**2.09**	**0.04**	0.05	0.36	0.72
Parks and green spaces	**0.22**	**8.05**	**0.00**	**0.35**	**4.10**	**0.00**
Hospitals	**0.15**	**5.15**	**0.00**	**0.30**	**3.69**	**0.00**
Religious sites	−0.19	−0.50	0.61	**0.38**	**3.89**	**0.00**
(Constant)	0.01	0.31	0.76	0.00	−0.14	0.89

^1^ Numbers in bold represent significant coefficients at the 0.01 level.

**Table 5:** Average activity duration in each type of activity place and percentage of participants that have at least once visited this type of activity place.

Activity Places	Average Activity Duration		Percentage of Participants	
	Hui Residents	Han Residents	Hui Residents	Han Residents
	min	min	%	%
Workplaces	471	423	53.5	63.6
Relatives’ home	62	46	16.7	16.2
Shops	62	46	23.7	39.4
Restaurants	12	36	7.9	21.5
Parks and green spaces	26	50	11.2	26.4
Hospitals	18	16	13.7	5.2
Religious sites	52	1	16.7	1.3
